# *N*-(2,4-Dimethyl­phen­yl)-2-methyl­benzamide

**DOI:** 10.1107/S1600536809009830

**Published:** 2009-03-25

**Authors:** B. Thimme Gowda, Miroslav Tokarčík, Jozef Kožíšek, Vinola Zeena Rodrigues, Hartmut Fuess

**Affiliations:** a Department of Chemistry, Mangalore University, Mangalagangotri 574 199, Mangalore, India; b Faculty of Chemical and Food Technology, Slovak Technical University, Radlinského 9, SK-812 37 Bratislava, Slovak Republic; c Institute of Materials Science, Darmstadt University of Technology, Petersenstrasse 23, D-64287 Darmstadt, Germany

## Abstract

In the title compound, C_16_H_17_NO, the N—H bond is in an *anti* conformation with respect to the C=O bonds. The aniline and benzoyl rings are almost coplanar, making a dihedral angle of 4.9 (3)°. The plane of the amide group makes an angle of 61.3 (3)° with the aniline ring and 58.3 (3)° with the benzoyl ring. In the crystal, the mol­ecules are linked by N—H⋯O hydrogen bonds into chains running along the *b* axis.

## Related literature

For related structures, see Gowda *et al.* (2003 ▶, 2008**a* ▶,b* ▶,*c* ▶).
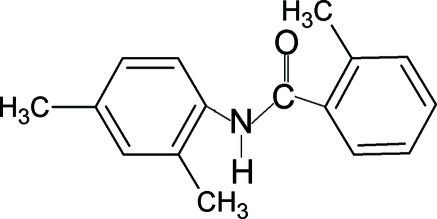


## Experimental

### 

#### Crystal data

C_16_H_17_NO
*M* *_r_* = 239.31
Orthorhombic, 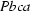

*a* = 6.0062 (4) Å
*b* = 9.8036 (6) Å
*c* = 44.943 (4) Å
*V* = 2646.4 (3) Å^3^
*Z* = 8
Mo *K*α radiation
μ = 0.08 mm^−1^
*T* = 295 K
0.53 × 0.48 × 0.12 mm

#### Data collection

Oxford Diffraction Xcalibur System diffractometer
Absorption correction: multi-scan (*CrysAlis RED*; Oxford Diffraction, 2008 ▶) *T*_min_ = 0.958, *T*_max_ = 0.991
20129 measured reflections
2467 independent reflections
1863 reflections with *I* > 2σ(*I*)
*R*_int_ = 0.073

#### Refinement

*R*[*F* ^2^ > 2σ(*F* ^2^)] = 0.098
*wR*(*F* ^2^) = 0.241
*S* = 1.14
2467 reflections
169 parameters
1 restraint
H atoms treated by a mixture of independent and constrained refinement
Δρ_max_ = 0.24 e Å^−3^
Δρ_min_ = −0.21 e Å^−3^

### 

Data collection: *CrysAlis CCD* (Oxford Diffraction, 2008 ▶); cell refinement: *CrysAlis RED* (Oxford Diffraction, 2008 ▶); data reduction: *CrysAlis RED*; program(s) used to solve structure: *SHELXS97* (Sheldrick, 2008 ▶); program(s) used to refine structure: *SHELXL97* (Sheldrick, 2008 ▶); molecular graphics: *ORTEP-3* (Farrugia, 1997 ▶) and *DIAMOND* (Brandenburg, 2002 ▶); software used to prepare material for publication: *SHELXL97*, *PLATON* (Spek, 2009 ▶) and *WinGX* (Farrugia, 1999 ▶).

## Supplementary Material

Crystal structure: contains datablocks I, global. DOI: 10.1107/S1600536809009830/bt2892sup1.cif

Structure factors: contains datablocks I. DOI: 10.1107/S1600536809009830/bt2892Isup2.hkl

Additional supplementary materials:  crystallographic information; 3D view; checkCIF report

## Figures and Tables

**Table 1:** Hydrogen-bond geometry (Å, °)

*D*—H⋯*A*	*D*—H	H⋯*A*	*D*⋯*A*	*D*—H⋯*A*
N1—H1*N*⋯O1^i^	0.90 (3)	1.99 (3)	2.880 (4)	170 (4)

Symmetry code: (i) 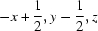.

**Fig. 1.**
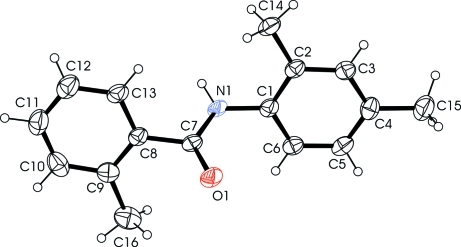
Molecular structure of the title compound showing the atom labelling scheme. The displacement ellipsoids are drawn at the 30% probability level. H atoms are represented as small spheres of arbitrary radii.

**Fig. 2.**
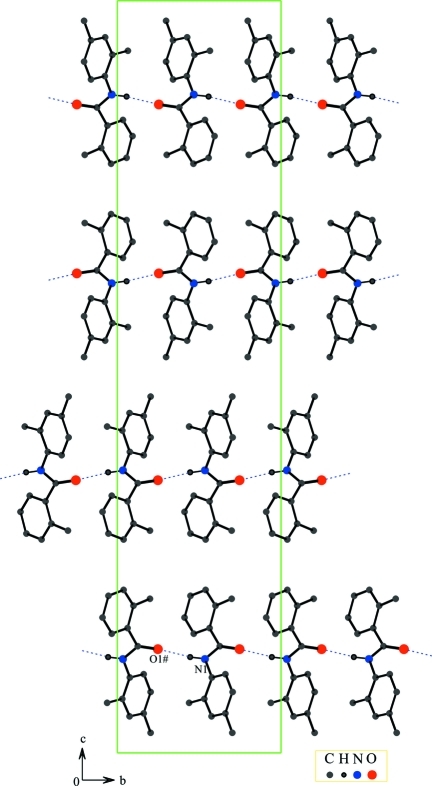
Crystal structure of the title compound viewed in the bc-plane. Chains running along the b axis generated by N—H···O# hydrogen bonds are shown by dashed lines. [Symmetry code (#): -x + 1/2, y - 1/2,z]. H atoms not involved in hydrogen bonding have been omitted.

## supplementary crystallographic information

### Comment

In the present work, as part of our study of substituent effects on the structures of benzanilides (Gowda *et al.*, 2003; 2008*a, b, c*), the structure of 2-methyl-*N*-(2,4-dimethylphenyl)benzamide has been determined. The N—H and C=O bonds in the amide segment are *anti* to each other (Fig. 1), similar to what is observed in 2-methyl-*N*-(phenyl)benzamide (Gowda *et al.*, 2008*a*), 2-methyl-*N*-(4-methylphenyl)benzamide (Gowda *et al.*, 2008*c*), 2-methyl-*N*-(2,6-dimethylphenyl)-benzamide (Gowda *et al.*, 2008*b*) and other benzanilides. Further, the conformation of the amide oxygen   is *syn* to the *ortho*-methyl group in the benzoyl ring and the amide hydrogen is *syn* to the *ortho*-methyl group in the aniline ring. The anilino and benzoyl rings are almost coplanar with a dihedral angle of 4.9 (3)°. The plane of the amide group makes the angles of 61.3 (3)° with the anilino ring and 58.3 (3)° with the benzoyl ring. A packing diagram of the title compound viewed in the *bc*-plane is shown in Fig. 2. Molecular chains running along the *b*-axis are generated by N—H···O hydrogen bonds (Table 1).

### Experimental

The title compound was prepared according to the method described by Gowda *et al.* (2003). The purity of the compound was checked by determining its melting point. It was characterized by recording its infrared and NMR spectra. Plate-like colourless single crystals of the title compound were obtained by slow evaporation from an ethanol solution (0.5 g in about 30 ml of ethanol) at room temperature.

### Refinement

H atoms attached to C atoms were placed in calculated positions and refined within a riding model with C—H distances of 0.93 or 0.96 Å. The coordinates of the H atom of the amide group were refined with a restraint of 0.86 (2)Å  for the H—N distance. The *U*_iso_(H) values were set at 1.2 *U*_eq_(C-aromatic, N) and 1.5 *U*_eq_(C_methyl_).

### Crystal data

**Table:**

C_16_H_17_NO	*F*(000) = 1024
*M**_r_* = 239.31	*D*_x_ = 1.201 Mg m^−^^3^
Orthorhombic, *P**b**c**a*	Mo *K*α radiation, λ = 0.71073 Å
Hall symbol: -P 2ac 2ab	Cell parameters from 7082 reflections
*a* = 6.0062 (4) Å	θ = 3.2–27.5°
*b* = 9.8036 (6) Å	µ = 0.08 mm^−^^1^
*c* = 44.943 (4) Å	*T* = 295 K
*V* = 2646.4 (3) Å^3^	Block, colourless
*Z* = 8	0.53 × 0.48 × 0.12 mm

### Data collection

**Table:**

Oxford Diffraction Xcalibur System diffractometer	2467 independent reflections
graphite	1863 reflections with *I* > 2σ(*I*)
Detector resolution: 10.434 pixels mm^-1^	*R*_int_ = 0.073
ω scans with κ offsets	θ_max_ = 25.7°, θ_min_ = 3.6°
Absorption correction: multi-scan (*CrysAlis RED*; Oxford Diffraction, 2008)	*h* = −7→7
*T*_min_ = 0.958, *T*_max_ = 0.991	*k* = −11→11
20129 measured reflections	*l* = −54→54

### Refinement

**Table:**

Refinement on *F*^2^	Primary atom site location: structure-invariant direct methods
Least-squares matrix: full	Secondary atom site location: difference Fourier map
*R*[*F*^2^ > 2σ(*F*^2^)] = 0.098	Hydrogen site location: inferred from neighbouring sites
*wR*(*F*^2^) = 0.241	H atoms treated by a mixture of independent and constrained refinement
*S* = 1.14	*w* = 1/[σ^2^(*F*_o_^2^) + (0.0735*P*)^2^ + 4.7656*P*] where *P* = (*F*_o_^2^ + 2*F*_c_^2^)/3
2467 reflections	(Δ/σ)_max_ < 0.001
169 parameters	Δρ_max_ = 0.24 e Å^−^^3^
1 restraint	Δρ_min_ = −0.20 e Å^−^^3^

### Special details

**Table:**

Experimental. CrysAlis RED (Oxford Diffraction, 2008). Empirical absorption correction using spherical harmonics, implemented in SCALE3 ABSPACK scaling algorithm.
Geometry. All e.s.d.'s (except the e.s.d. in the dihedral angle between two l.s. planes) are estimated using the full covariance matrix. The cell e.s.d.'s are taken into account individually in the estimation of e.s.d.'s in distances, angles and torsion angles; correlations between e.s.d.'s in cell parameters are only used when they are defined by crystal symmetry. An approximate (isotropic) treatment of cell e.s.d.'s is used for estimating e.s.d.'s involving l.s. planes.
Refinement. Refinement of *F*^2^ against ALL reflections. The weighted *R*-factor *wR* and goodness of fit *S* are based on *F*^2^, conventional *R*-factors *R* are based on *F*, with *F* set to zero for negative *F*^2^. The threshold expression of *F*^2^ > σ(*F*^2^) is used only for calculating *R*-factors(gt) *etc*. and is not relevant to the choice of reflections for refinement. *R*-factors based on *F*^2^ are statistically about twice as large as those based on *F*, and *R*- factors based on ALL data will be even larger.

### Fractional atomic coordinates and isotropic or equivalent isotropic displacement parameters (Å^2^)

**Table:**

	*x*	*y*	*z*	*U*_iso_*/*U*_eq_	
C1	0.4332 (7)	0.5763 (4)	0.09991 (8)	0.0446 (9)	
C2	0.3831 (7)	0.5294 (4)	0.07156 (9)	0.0470 (9)	
C3	0.5158 (7)	0.5746 (4)	0.04829 (9)	0.0501 (10)	
H3	0.4813	0.5463	0.0291	0.06*	
C4	0.6975 (7)	0.6600 (5)	0.05232 (9)	0.0557 (11)	
C5	0.7436 (7)	0.7029 (4)	0.08089 (9)	0.0543 (10)	
H5	0.8635	0.761	0.0843	0.065*	
C6	0.6148 (7)	0.6611 (4)	0.10447 (9)	0.0501 (10)	
H6	0.6498	0.6898	0.1236	0.06*	
C7	0.1870 (7)	0.6237 (3)	0.14167 (8)	0.0442 (9)	
C8	0.0506 (6)	0.5613 (4)	0.16578 (8)	0.0442 (9)	
C9	0.0815 (7)	0.5979 (4)	0.19532 (9)	0.0533 (10)	
C10	−0.0551 (9)	0.5371 (5)	0.21657 (10)	0.0712 (14)	
H10	−0.0356	0.5587	0.2366	0.085*	
C11	−0.2191 (9)	0.4454 (5)	0.20853 (12)	0.0743 (14)	
H11	−0.311	0.4081	0.2231	0.089*	
C12	−0.2471 (9)	0.4092 (5)	0.17960 (11)	0.0711 (13)	
H12	−0.3568	0.3468	0.1743	0.085*	
C13	−0.1125 (7)	0.4655 (4)	0.15833 (10)	0.0542 (10)	
H13	−0.1298	0.4396	0.1386	0.065*	
C14	0.1901 (7)	0.4363 (4)	0.06628 (10)	0.0589 (11)	
H14A	0.1679	0.4252	0.0453	0.088*	
H14B	0.0585	0.4749	0.075	0.088*	
H14C	0.2199	0.3492	0.0752	0.088*	
C15	0.8378 (9)	0.7033 (5)	0.02654 (11)	0.0769 (15)	
H15A	0.9908	0.7082	0.0326	0.115*	
H15B	0.7899	0.7913	0.0197	0.115*	
H15C	0.823	0.6381	0.0107	0.115*	
C16	0.2558 (10)	0.6978 (5)	0.20471 (10)	0.0754 (14)	
H16A	0.4004	0.6625	0.1999	0.113*	
H16B	0.2459	0.7124	0.2258	0.113*	
H16C	0.2331	0.7827	0.1945	0.113*	
N1	0.3000 (6)	0.5350 (3)	0.12468 (7)	0.0491 (9)	
H1N	0.287 (7)	0.445 (3)	0.1279 (8)	0.059*	
O1	0.1884 (5)	0.7473 (3)	0.13756 (6)	0.0638 (9)	

### Atomic displacement parameters (Å^2^)

**Table:**

	*U*^11^	*U*^22^	*U*^33^	*U*^12^	*U*^13^	*U*^23^
C1	0.046 (2)	0.0306 (18)	0.057 (2)	0.0064 (17)	0.0054 (18)	0.0037 (16)
C2	0.043 (2)	0.034 (2)	0.064 (2)	0.0031 (17)	0.0023 (18)	0.0020 (17)
C3	0.052 (2)	0.042 (2)	0.057 (2)	0.001 (2)	−0.0008 (19)	−0.0006 (17)
C4	0.045 (2)	0.058 (3)	0.064 (2)	0.001 (2)	0.0058 (19)	0.010 (2)
C5	0.043 (2)	0.041 (2)	0.079 (3)	−0.0051 (19)	−0.001 (2)	0.002 (2)
C6	0.047 (2)	0.042 (2)	0.060 (2)	0.0015 (18)	−0.0027 (19)	−0.0006 (18)
C7	0.048 (2)	0.0267 (18)	0.058 (2)	0.0048 (17)	−0.0024 (18)	0.0022 (15)
C8	0.045 (2)	0.0311 (18)	0.056 (2)	0.0058 (17)	0.0021 (18)	0.0040 (16)
C9	0.055 (3)	0.041 (2)	0.063 (2)	0.013 (2)	−0.003 (2)	0.0011 (18)
C10	0.088 (4)	0.070 (3)	0.056 (2)	0.025 (3)	0.012 (3)	0.002 (2)
C11	0.073 (3)	0.059 (3)	0.091 (4)	0.006 (3)	0.033 (3)	0.012 (3)
C12	0.056 (3)	0.061 (3)	0.096 (4)	−0.004 (2)	0.014 (3)	0.004 (3)
C13	0.060 (3)	0.032 (2)	0.071 (3)	0.001 (2)	0.004 (2)	0.0011 (18)
C14	0.053 (3)	0.044 (2)	0.080 (3)	−0.013 (2)	0.004 (2)	−0.005 (2)
C15	0.069 (3)	0.077 (3)	0.085 (3)	−0.017 (3)	0.015 (3)	0.011 (3)
C16	0.082 (3)	0.076 (3)	0.068 (3)	0.010 (3)	−0.021 (3)	−0.006 (2)
N1	0.064 (2)	0.0178 (15)	0.0659 (19)	0.0033 (15)	0.0112 (17)	0.0033 (13)
O1	0.080 (2)	0.0284 (14)	0.083 (2)	0.0061 (15)	0.0167 (17)	0.0030 (13)

### Geometric parameters (Å, °)

**Table:**

C1—C6	1.387 (5)	C9—C16	1.494 (7)
C1—C2	1.388 (5)	C10—C11	1.382 (7)
C1—N1	1.430 (5)	C10—H10	0.93
C2—C3	1.388 (5)	C11—C12	1.358 (7)
C2—C14	1.494 (5)	C11—H11	0.93
C3—C4	1.387 (6)	C12—C13	1.369 (6)
C3—H3	0.93	C12—H12	0.93
C4—C5	1.380 (6)	C13—H13	0.93
C4—C15	1.494 (6)	C14—H14A	0.96
C5—C6	1.375 (5)	C14—H14B	0.96
C5—H5	0.93	C14—H14C	0.96
C6—H6	0.93	C15—H15A	0.96
C7—O1	1.225 (4)	C15—H15B	0.96
C7—N1	1.342 (5)	C15—H15C	0.96
C7—C8	1.490 (5)	C16—H16A	0.96
C8—C9	1.388 (5)	C16—H16B	0.96
C8—C13	1.398 (5)	C16—H16C	0.96
C9—C10	1.393 (6)	N1—H1N	0.90 (3)
			
C6—C1—C2	120.3 (4)	C12—C11—C10	120.6 (5)
C6—C1—N1	119.7 (3)	C12—C11—H11	119.7
C2—C1—N1	120.0 (4)	C10—C11—H11	119.7
C3—C2—C1	117.5 (4)	C11—C12—C13	119.3 (5)
C3—C2—C14	121.4 (4)	C11—C12—H12	120.3
C1—C2—C14	121.1 (4)	C13—C12—H12	120.3
C4—C3—C2	123.1 (4)	C12—C13—C8	121.2 (4)
C4—C3—H3	118.4	C12—C13—H13	119.4
C2—C3—H3	118.4	C8—C13—H13	119.4
C5—C4—C3	117.6 (4)	C2—C14—H14A	109.5
C5—C4—C15	121.5 (4)	C2—C14—H14B	109.5
C3—C4—C15	120.9 (4)	H14A—C14—H14B	109.5
C6—C5—C4	120.9 (4)	C2—C14—H14C	109.5
C6—C5—H5	119.6	H14A—C14—H14C	109.5
C4—C5—H5	119.6	H14B—C14—H14C	109.5
C5—C6—C1	120.5 (4)	C4—C15—H15A	109.5
C5—C6—H6	119.7	C4—C15—H15B	109.5
C1—C6—H6	119.7	H15A—C15—H15B	109.5
O1—C7—N1	123.5 (4)	C4—C15—H15C	109.5
O1—C7—C8	121.3 (3)	H15A—C15—H15C	109.5
N1—C7—C8	115.2 (3)	H15B—C15—H15C	109.5
C9—C8—C13	119.8 (4)	C9—C16—H16A	109.5
C9—C8—C7	121.0 (4)	C9—C16—H16B	109.5
C13—C8—C7	119.2 (3)	H16A—C16—H16B	109.5
C8—C9—C10	117.8 (4)	C9—C16—H16C	109.5
C8—C9—C16	122.3 (4)	H16A—C16—H16C	109.5
C10—C9—C16	120.0 (4)	H16B—C16—H16C	109.5
C11—C10—C9	121.3 (4)	C7—N1—C1	122.8 (3)
C11—C10—H10	119.4	C7—N1—H1N	120 (3)
C9—C10—H10	119.4	C1—N1—H1N	117 (3)
			
C6—C1—C2—C3	−2.3 (5)	N1—C7—C8—C13	−57.5 (5)
N1—C1—C2—C3	179.0 (3)	C13—C8—C9—C10	−0.4 (6)
C6—C1—C2—C14	179.1 (4)	C7—C8—C9—C10	178.8 (4)
N1—C1—C2—C14	0.4 (5)	C13—C8—C9—C16	178.9 (4)
C1—C2—C3—C4	2.0 (6)	C7—C8—C9—C16	−1.9 (6)
C14—C2—C3—C4	−179.4 (4)	C8—C9—C10—C11	−1.3 (6)
C2—C3—C4—C5	−1.2 (6)	C16—C9—C10—C11	179.4 (4)
C2—C3—C4—C15	178.7 (4)	C9—C10—C11—C12	1.8 (7)
C3—C4—C5—C6	0.7 (6)	C10—C11—C12—C13	−0.6 (7)
C15—C4—C5—C6	−179.2 (4)	C11—C12—C13—C8	−1.1 (7)
C4—C5—C6—C1	−1.0 (6)	C9—C8—C13—C12	1.6 (6)
C2—C1—C6—C5	1.9 (6)	C7—C8—C13—C12	−177.6 (4)
N1—C1—C6—C5	−179.4 (3)	O1—C7—N1—C1	−0.2 (6)
O1—C7—C8—C9	−58.6 (5)	C8—C7—N1—C1	177.8 (4)
N1—C7—C8—C9	123.3 (4)	C6—C1—N1—C7	62.0 (5)
O1—C7—C8—C13	120.6 (4)	C2—C1—N1—C7	−119.3 (4)

### Hydrogen-bond geometry (Å, °)

**Table:**

*D*—H···*A*	*D*—H	H···*A*	*D*···*A*	*D*—H···*A*
N1—H1N···O1^i^	0.90 (3)	1.99 (3)	2.880 (4)	170 (4)

Symmetry codes: (i) −*x*+1/2, *y*−1/2, *z*.
